# Quality of life and BMI changes in youth participating in an integrated pediatric obesity treatment program

**DOI:** 10.1186/1477-7525-11-116

**Published:** 2013-07-10

**Authors:** Keeley J Pratt, Suzanne Lazorick, Angela L Lamson, Andrada Ivanescu, David N Collier

**Affiliations:** 1 Department of Human Development and Family Science, The Ohio State University, 109C Campbell Hall, 1787 Neil Ave, Columbus, OH 43210, USA; 2 Departments of Pediatrics and Public Health, Brody School of Medicine, East Carolina University, Greenville, NC 27858, USA; 3 Department of Child Development and Family Relations, College of Human Ecology, East Carolina University, Greenville, NC 27858, USA; 4 Department of Biostatistics, College of Allied Health Sciences, East Carolina University, Greenville, NC 27858, USA

**Keywords:** Pediatric obesity, Quality of life, Depression, Obesity treatment

## Abstract

**Background:**

Changes in Quality of Life (QOL) measures over time with treatment of obesity have not previously been described for youth. We describe the changes from baseline through two follow up visits in youth QOL (assessed by the Pediatric Quality Life Inventory, PedsQL4.0), teen depression (assessed by the Patient Health Questionnaire, PHQ9A), Body Mass Index (BMI) and BMI z-score. We also report caregiver proxy ratings of youth QOL.

**Methods:**

A sample of 267 pairs of youth and caregiver participants were recruited at their first visit to an outpatient weight-treatment clinic that provides care integrated between a physician, dietician, and mental health provider; of the 267, 113 attended a visit two (V2) follow-up appointment, and 48 attended visit three (V3). We investigated multiple factors longitudinally experienced by youth who are overweight and their caregivers across up to three different integrated care visits. We determined relationships at baseline in QOL, PHQ9A, and BMI z-score, as well as changes in variables over time using linear mixed models with time as a covariate.

**Results:**

Overall across three visits the results indicate that youth had slight declines in relative BMI, significant increases in their QOL and improvements in depression.

**Conclusions:**

We encourage clinicians and researchers to track youth longitudinally throughout treatment to investigate not only youth’s BMI changes, but also psychosocial changes including QOL.

## Introduction

Childhood obesity is identified as a nationwide epidemic that affects youth regardless of gender, age, race or ethnic group [1-3]. Current statistics show that 31.8% of youth between the ages of 2 to 19 are diagnosed as overweight (having a BMI above the 85th percentile for their sex and age) and 16.9% are diagnosed as obese (having a BMI above the 95th percentile for their sex and age) [3]. This epidemic has prompted development of a set of recommendations for treating obesity in youth. The report, entitled Expert Committee Recommendations Regarding the Prevention, Assessment, and Treatment of Child and Adolescent Overweight and Obesity [4] summarizes the findings of the currently accepted practices for childhood obesity prevention, assessment, intervention, and treatment [1]. The report details four stages of childhood obesity treatment: 1) prevention plus; 2) structured weight management; 3) comprehensive, multidisciplinary intervention, and 4) tertiary care intervention [1]. These recommendations incorporate several elements such as family involvement and the inclusion of multidisciplinary providers in order to provide optimal biopsychosocial care for youth and families [5]. Clinically, for youth who have been gaining weight up until they initiate treatment, decreases in weight gain, leveling of current weight, and subsequent weight loss are all considered successes. These “successes” are considered clinically significant as progress in treatment, even though over a short time period they may not be statistically significant or yet large enough yet to impart substantial decrease in health risk.

One method to comprehensively evaluate how weight may impact youth both biologically (or physically) and psychosocially is to formally assess quality of life (QOL) using standardized inventories such as the Pediatric Quality of Life Inventory™ (PedsQL). The PedsQL inventory is used to assess the respondent’s quality of life by measuring physical, emotional, social, and school functioning, thus providing physical and psychosocial outcomes in one tool. The PedQL is used both as a research assessment and a clinical tool for treatment teams who are working with overweight youth. Clinically, it is important to measure QOL in both the overweight youth and their caregiver since often perceptions differ regarding how the youth is doing physically, emotionally, socially, and in school. Providers can talk about the discrepancy in youth and caregiver QOL reports to gauge more accurately where to focus efforts and goals. Often youth have a difficult time talking about areas of QOL where they may have impairments; the PedQL can be a tool to note specific areas where the treatment team may be able to focus on goals or make appropriate referrals. For example, if a youth mentions they often feel down or low (emotional domain), the treatment team can discuss the youth’s response, get their caregiver’s perspective of the youth’s emotional health, and determine how to incorporate or refer out to address this issue. When investigating the relationship between obesity and QOL in youth some researchers have concluded that there is not an impaired QOL [6], but most report that QOL is inversely related to weight. As a youth’s weight increases, his or her QOL decreases, so the most overweight youth have the most significantly impaired QOL [7-11]. Schwimmer et al. found that obese youth are 5.5 times more likely than healthy youth to have impaired QOL, making QOL for an obese youth similar to that of a youth diagnosed with cancer [8].

In addition to impaired QOL, it is known that youth who are obese have increased likelihood for psychological problems that may persist into adulthood as compared to youth who are not obese [12]. Overweight treatment seeking youth report more depressive symptoms compared with their normal weight and non treatment seeking peers [13]. Obese youth with increased depressive symptoms reported lower QOL [11]. Due to the lack of longitudinal data, it is unclear how specific psychological issues (e.g., youth and maternal depression) influence QOL over time and/or with treatment for obesity. This exploratory study follows a cohort of obese youth and their caregivers during treatment and assesses for changes in and associations with QOL, depression and body mass index (BMI) z-score.

### Purpose

We sought to understand further how patient QOL and depression change over time with treatment for obesity. Our purpose is to describe the changes during treatment from youth and caregiver baseline variables in QOL, teen depression status, and youth (child/teen) BMI and BMI z-score from baseline through two follow-up visits.

## Methods

### Setting

The Pediatric Healthy Weight Research and Treatment Center (PHWRTC) provides comprehensive multidisciplinary intervention to youth referred by their primary care providers because of a concern about the youth’s weight and the risk of weight-related comorbidities. In addition to employing a stage two-treatment strategy [4], the care team uses an integrated care model, which is an intense form of collaborative multidisciplinary care. Care is highly coordinated between medical and mental health providers, as evidenced by shared treatment plans [14]. Providers include two different physicians that rotate clinical time, one registered dietitian, one doctoral level medical family therapist, and one master’s level family therapy intern. At each visit, patients and their caregivers meet with the physician and mental health provider together and a dietician who all work from an integrated care model. Height, weight, BMI and blood pressure are tracked by the nursing staff, and BMI percentile is plotted by the medical provider at each visit. Physical activity behaviors are tracked by the pediatrician at each visit and QOL and depression are tracked by the family therapist. Regular follow up appointments are scheduled, typically at least every three months, and ideally every month; however, the rural population, low income, and poor access to transportation thwart strong retention and high visit frequency.

### Participants

Youth ages 8–18 years and their caregivers were recruited for this study at the initial visit to the PHWRTC. Exclusion criteria included age less than eight years, cognitive impairment preventing research measures, or non-English speaking (approximately 1% of the patient population). This study included 267 youth and their caregivers, who initiated treatment and had two follow-up visits from July 2007 through November 2009.

### Study design

A longitudinal design is used to examine factors associated with weight-related outcomes (BMI z-score changes) experienced by youth and their caregivers across up to three consecutive integrated care visits (V1-baseline, V2, and V3). The sample for the study was a convenience sample at each wave of data collection. Ten families refused to participate due mainly due to time limitations at the initial appointment. This study included 267 youth and associated. Of these 267 pairs, 113 (42% of the original group) returned for a second visit (V2), and 48 (18% of the original group) returned for a third visit (V3) during the study timeframe. In our study the median was170 days (n = 48) between V1 and V3. There were more days that elapsed between V2 and V3 (n = 48; median = 87), as compared to days between V1 and V2 (n = 113; median = 77). Of the 267 youth, there were 147 (55.1%) children 8–12 years old and 120 (44.9%) teens that were 13–18 years old. This study was approved by the University Medical Center Institutional Review Board (approval number 08–0418).

### Measures

Youth participants’ date of birth, height and weight at each visit was extracted by the investigators from the medical chart. A demographic questionnaire was administered to the caregiver at the initial visit that queried race, age, gender, and family structure. Youth were categorized into two groups by age: 8–12 (child) and 13–18 (teen) years, to fit the recommended ages for the validated QOL and depression inventories. Race was categorized into three groups: black, white, and other.

At every visit research inventories administered to the patient and the caregiver included the age appropriate Pediatric Quality of Life Inventory (PedsQL4.0, 8–12 and 13–18 years old) and the Patient Health Questionnaire©, adolescent version (PHQ-9A; 13–18 years old) [15]. If questions arose while the youth or caregiver was taking the survey, a member of the research team was available to provide assistance or answer questions. Also, if needed, a member of the research team was available to assist youth who had trouble reading.

The PedsQL4.0 has been cited in numerous publications on childhood obesity [16-20]. Schwimmer and colleagues found that the total scale score for both the youth and caregiver reports have demonstrated at least a Cronbach α reliability coefficient of .90, and thus can be utilized for individual patient analysis and as a health related quality of life outcome measure for clinical trials [8]. Our sample yielded reliability coefficients of .89 and .92 for the PedsQL youth and caregiver, respectively. The PedsQL4.0 modules consist of 23 items, which are broken down into four dimensions of functioning: physical, emotional, social, and school. Items are ranked on a Likert scale ranging from (0) never a problem, (1) almost never a problem, (2) sometimes a problem, (3) often a problem, to (4) almost always a problem; but are scored reversely (i.e., 0 – never a problem = 100 and 4 – almost always a problem = 0). Per this measure, a higher score indicates a better QOL, whereas a lower score indicates a more impaired QOL. The following outcome variables were used for the PedsQL: youth total score, caregiver total score, youth subscale (physical, emotional, social and school) scores, and caregiver subscale scores.

The PHQ9A was used to assess depressive symptoms for adolescents, experienced throughout the two-week time frame immediately prior to the visit. The PHQ9A consists of nine questions, with responses ranging from: not at all (0), several days (1), more than half the days (2), and nearly every day (3). The result from the PHQ9A is a depression severity score, ranging from no depression (0–4), mild depression (5–9), moderate depression (10–14), moderately severe depression (15–19), and severe depression (20–27). This measure was used to assess for depression and suicidal ideation in teens (≥13) [21]. Kroenke, Spitzer, and Williams (2001) reviewed their earlier studies on the PHQ9 and PHQ9A [22,23] and reported a Cronbach’s α of 0.89 in internal reliability as well as excellent test-retest reliability [23]. For our sample, the PHQ9A reliability coefficients was .78 for teens.

We used BMI z-score (the LMS method) to assess for change in weight status over time. The LMS method converts a regular BMI measure to a normally distributed standard deviation, also known as a z-score [24]. BMI z-score is most helpful in identifying where an individual is relative to the population norm [25]. We used standard Centers for Disease Control growth charts for gender and age to determine weight category: overweight (85th ≤ 95th percentile), obese (95 ≤ 99th), and severely obese (≥ 99th) [26,27].

### Analysis

IBM SPSS version 18.0 and Matlab version 7.8.0 (R2009a) were used for analyses. Cronbach’s α was used to estimate the internal-consistency reliability of the PedsQL and PHQ9 inventories (see above scale-specific reliability coefficients). Associations between categorical variables were analyzed using a chi-square test for independence. One-way ANOVA was used to compare means between independent groups, and a paired-*t* test to compare within-group mean differences between visits. Pearson correlations were used to investigate relationships between QOL and depressive symptoms.

For the longitudinal analysis, we analyzed the relationships over time using linear mixed models [28] with time as a covariate, considering a model with random intercepts and slopes, and having an unstructured covariance structure. In the longitudinal analysis, one outlier was removed, since the time between consecutive visits (visit 1 and 2) was extremely large at 612 days. Patient trajectories are shown together with the estimated mean function (bold curve) obtained using a kernel smoother (a nonparametric function estimation procedure) with bandwidth chosen via the generalized cross-validation (GVC) procedure implemented in PACE package in Matlab.

## Results

The characteristics of youth and caregivers who comprised the sample are detailed in Table 1. The majority of the youth were female (54%), black (63%), and under 13 years of age (57%). The mean BMI of the youth sample at the initial visit was 37.3 (range 19.6-72.6); the mean BMI z-score was 2.5 (range 1.2-3.6). Over 72% of the youth were classified as severely obese at their initial visit with a median BMI of 36. Less than half of the youth were from two-parent families. Youth weight categories included: 1.9% overweight (n = 5), 25.5% obese (n = 68) and 72.7% severely obese (n = 194). Given the study focus on obesity, we excluded the overweight youth from longitudinal analyses.

**Table 1:** Sample characteristics at each visit

	***Visit 1***	***Visit 2***	***Visit 3***
	***(N = 267)***	***(N = 113)***	***(N = 48)***
	***Youth Demographics N (%)***		
***Gender***			
**Male**	122 (45.7)	44 (38.9)	20 (41.7)
**Female**	145 (54.3)	69 (61.1)	28 (58.3)
***Race***			
**White**	80 (30.0)	39 (34.5)	20 (41.7)
**Black**	169 (63.3)	70 (61.9)	28 (58.3)
**Other**	18 (6.7)	4 (3.6)	0
	***Anthropometric Data mean (SD)***		
***Body Mass Index (BMI)***	37.8(12.2)	38.2(8.7)	38.9(9.3)
***BMI z-score***	2.50(.34)	2.52(.33)	2.53(.40)
***BMI Category N (%)***			
**Overweight**	5 (1.9)	0	1 (2.1)
**Obese**	68 (25.5)	39 (26.5)	11 (22.9)
**Severely Obese**	194 (72.7)	83 (73.5)	36 (75.0)
	***Family Structure N (%)***		
**Two-parent**	128 (47.9)		
**Non-two parent**	139 (52.1)		

Additional demographic variables of caregivers were collected that are not shown in Table 1. Over 85% of the caregivers at the initial visit were the youth’s mothers. A majority of the caregivers were black (65%), had an associate degree, some college or a college degree (62%), and were employed (69%). The median age of the caregivers was 39, with ages ranging from 25 to 69. Less than 17% of the participants received traditional health insurance, and almost half received Medicaid.

### Quality of life

For the total group of youth with one outlier removed (n = 267), the mean total score for the PedsQL was 73.0 (range 19–100, with 100 equaling the highest QOL score possible) with a standard deviation (SD) of 15.0. The psychosocial mean score was 72.0 (range 10–100) with a SD of 17.6. For the individual functioning subscales: the physical mean was 77.2 (range 25–100) with a SD of 15.7, emotional mean was 72.1 (range 10–100) with a SD of 21.7, social mean was 71.9 (range 0–100) with a SD of 22.2, and school mean was 70.0 (range 0–100) with a SD of 18.9.

For the total group of caregivers (n = 267) the mean total score for the PedsQL on their proxy report of the youth’s functioning was 66.2 (range 14–100) with a SD of 18.2. The psychosocial mean score was 66.7 (range 17–100) with a SD of 18.9. For the individual functioning subscales: the physical mean was 65.38 (range 0–100) with a SD of 21.2, emotional mean was 68.0 (range 10–100) with a SD of 21.9, social mean was 65.4 (range 4–100) with a SD of 22.9, and school mean was 67.3 (range 0–100) with a SD of 21.9.

The full report of youth and caregiver PedsQL congruence at V1 has been reported previously with comparisons to reference data from youth who are a healthy weight [29].

### Depression

The mean depression score for all teens (n = 114) was 5.7 (range 0–19) with a SD of 4.5. When categorized by severity of mild (score ≤ 9) and moderate depression (score ≥ 10), 90 teens (78.9%) had a score ≤ 9 and 24 (21.1%) had a score ≥10.

### Changes over time

**Figure 1:**
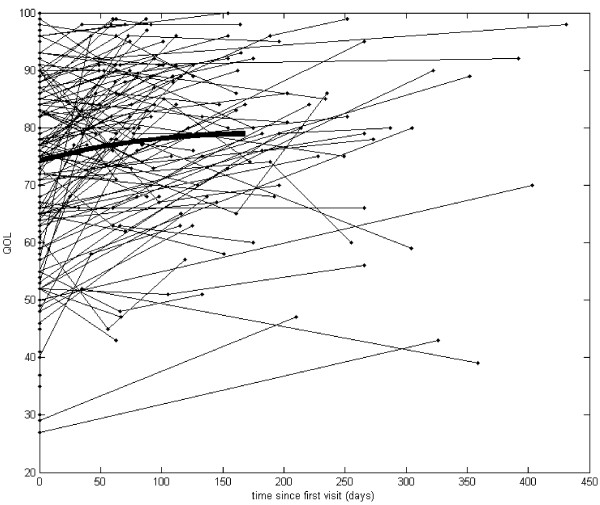
**Quality of life for all youth (age 8–18) participants (n = 266).** Compared to the average QOL of 74.28 at the first visit for all youth participants, the mean QOL increased by .034 points for each additional day all youth participants continue in the study.

**Figure 2:**
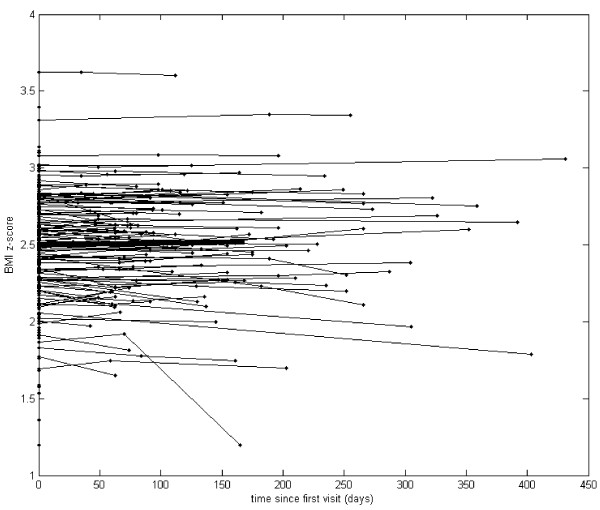
**Body mass index z-score for all youth (age 8–18) participants (n = 266).** Mixed model analysis indicates that the mean BMI z-score significantly decreased over time. Compared to the average BMI z-score 2.49 at the first visit, the mean BMI z-score decreased by .00011 for each additional day the participants continue in the study.

**Figure 3:**
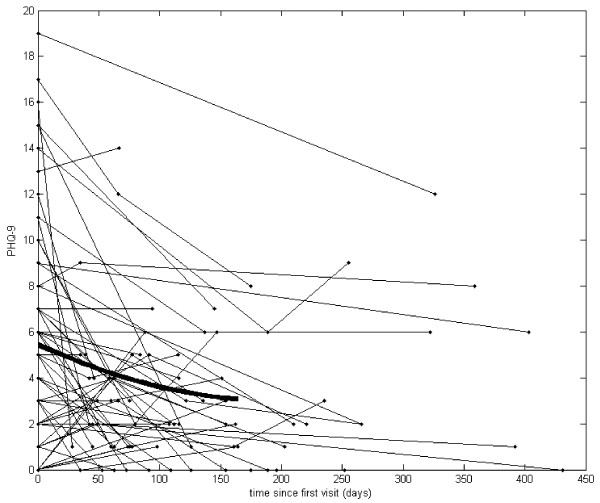
**Patient Healthcare Questionnaire (PHQ) for all teen (age 13–18) participants.** Compared to the average PHQ of 5.35 for teens at the first visit, the mean PHQ decreased by .01 points for each additional day the participants continue in the study.

**Figure 4:**
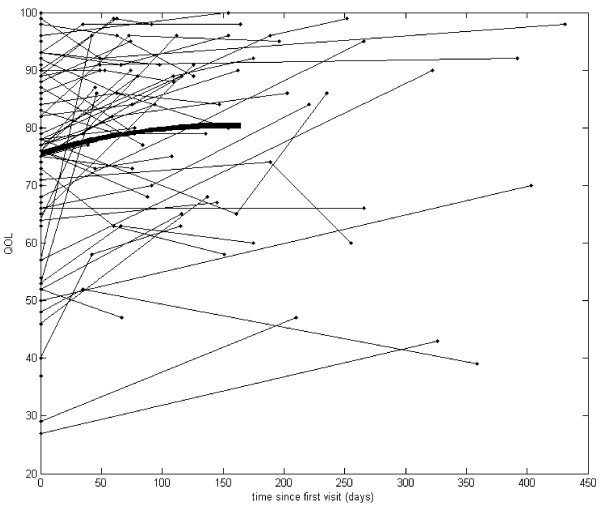
**Quality of life for all teen (age 13–18) participants.** Compared to the average QOL of 75.40 at the first visit, the mean QOL increased by .0335 points for each additional day the participants continued in the study.

Our analysis started with graphs of the actual observations recorded over time (see Figures 1, 2, 3 and 4). Due to the sparsely distributed data, first we chose to visually depict our results over time [30] and estimate the mean function [30]. The raw observed trajectories for each patient are presented in Figures 1, 2, 3 and 4. Our aim was to visually display the changes over time in QOL for all youth (Figure 1), BMI z-score for all youth (Figure 2), QOL for teen (Figure 3) and PHQ9A for teen (Figure 4) are shown with the estimated mean function (bold curve). In addition, we estimated the overall expected trajectory for the entire sample using the mean function to reveal the anticipated changes over time for BMI z-score, QOL, and PHQ9A. The estimated mean functions are shown in the figures as a bolded curve. This estimated mean function was obtained using a statistical procedure developed for sparsely distributed data [30] and uses a local kernel smoother approach. The mean function for the range of observed days was plotted for the first part of the time range because we had more observed data points. Overall, there was an increase in QOL, decrease in PHQ9A, and very slight decrease in BMI z-score over the duration of the study from V1 to V3 (or from zero to 450 days).

We further considered linear effects in the mixed model data analysis by estimating random slopes for each patient and then obtaining the estimated average slope to describe the observed effect of QOL, PHQ9A, and BMI z-score over time for the entire sample. The average QOL intercept across all youth participants was 74.28 (t (260) = 78.9, p < .001), and the average slope was .034 (t (61) = 4.9, p < .001). Hence, we found that compared to the average QOL of 74.28 at the first visit, the mean QOL increased by .034 points for each additional day all youth participants continue in the study. Mixed model analysis indicates that the mean BMI z-score significantly decreased over time. The average intercept across all youth participants was 2.49 (t (264.0) = 120.1, p < .001), and the average slope was −0.00011 (t (66.3) = −2.54, p = .013). We found that compared to the average BMI z-score 2.49 at the first visit, the mean BMI z-score decreased by .00011 for each additional day the participants continue in the study.

Mixed model analysis for teens showed that the average QOL intercept across participants was 75.40 (t (117) = 53.3, p < .001), and the average slope was .0335 (t (27) = 3.6, p = .001). Hence, we found that compared to the average QOL of 75.40 at the first visit, the mean QOL increased by .0335 points for each additional day the participants continued in the study. Mixed model analysis results for teens relating time (or days between visits), as explanatory variable, and PHQ9A, as response variable, showed that the average PHQ9A intercept across participants was 5.35 (t (121) = 14.2, p < .001), and the average slope was -.01 (t (56) = −4.2, p < .001). Hence, we found that compared to the average PHQ9A of 5.35 for teens at the first visit, the mean PHQ9A decreased by .01 points for each additional day the participants continue in the study.

We also wanted to investigate if, for teens, QOL is a significant predictor for PHQ9A in addition to the time since first visit. Mixed model analysis results show that both time (estimate = −0.007, t (22.3) = −3.19, p = .004) and QOL over time (estimate = −0.18, t (118.6) = −13.7, p < .001) were significant; so an increase in QOL over time yielded a significant decrease in mean PHQ9A over time.

After assessing if PHQ9A predicts QOL over time, as described above, we next determined if QOL and time were significant predictors for mean change in PHQ9A. Both time (estimate = −0.007, t (22.3) = −3.19, p = .004) and QOL over time (estimate = −0.18, t (118.6) = −13.7, p < .001) were significant; so an increase in QOL over time yields a significant decrease in mean PHQ9A over time.

## Discussion

In order to explore longitudinal changes, we assessed QOL, depressive symptoms, and BMI z-score indicators for overweight youth over time. Overall, across three visits (V1-V2-V3), our results indicated youth’s BMI z-score decreased slightly, their QOL significantly increased, and teen depression level improved. Likewise, caregivers’ perception of their youth’s QOL increased across three visits.

Interestingly, youth from V1 to V2 and V1 toV3 had significant improvements in their QOL, despite their BMI z-score and the majority of our sample being either obese or severely obese. This is especially important to consider, given that other researchers have reported that quality of life is inversely related to weight; as a youth’s weight increased, his/her QOL decreased, suggesting that the most overweight youth have the most significantly impaired QOL [13]. In a cross-sectional study, Williams et al. [10] compared youth of different BMI categories (normal, overweight, obese). In that research, obese youth were found to have a lower QOL than their normal and overweight peers. However, previous longitudinal research focusing on differences between youth in different severity categories of obesity (i.e., obese vs. severely obese) has not been done; our results indicate that even those who are most obese (severely obese or ≥ 99th percentile) had positive results in QOL with treatment, even with only modest improvements in BMI and no change in weight category.

After adjusting for time, improvement in teen depression (PHQ9A score) was strongly related to QOL improvement. This result emphasizes potential value in assessing for depression (in the teen and caregiver) in tandem with a QOL inventory. While past researchers have assessed for youth or caregiver depression or for QOL [11], none published to date have assessed for youth depression longitudinally in tandem with a QOL inventory. In light of our observed significant association between QOL and PHQ9A, our results suggest that with treatment using an integrated model emphasizing both physical and psychosocial factors, in obese youth both QOL and depression can improve even when BMI change is modest. In childhood obesity treatment, BMI improvement is ultimately the goal; however after youth make improvements in QOL and depression they may be more able and confident to adopt and work toward goals that result in weight-loss.

### Limitations

This study has several limitations. Previously researchers have demonstrated differences in treatment seeking vs. non-treatment seeking youth in QOL and on other measures of psychosocial health [13,15]. Thus, our results are best applied to those youth and families who are seeking treatment. Secondly, our sample was collected from one site in one specific location. Based on our population, the generalizabilty of our findings may only apply to those youth similar to our sample who are English speaking, and obese or severely obese, rather than overweight.

Although it was not our purpose, we looked for predictors of attending (or not attending) a follow-up and did not find any significant predictor based on race, BMI category, weight loss, gender, or age. Additionally, we only assessed depression in teens, not in youth under 12 years of age. We believe that our convenience sample, which was followed for up to two years, accurately reflects childhood obesity treatment attrition, whereby national attrition rates are estimated to be between 27% and 73% and half of participating families in treatment programs drop out of treatment [31,32].

We also only analyzed youth-caregiver dyads, which may neglect other important family members that are important in the youth’s daily life (e.g., grandparents, teachers, siblings, etc.). Finally, we only report data for up to three visits with relatively long time periods between each visit, in part due to the rural community, and the frequency (typically monthly) at which visits can be offered at the clinic. Thus different results might be seen in settings that can see patients more frequently (such as stage three treatment facilities that are recommended to see patients weekly for “intensive” treatment). Given that our results suggest improvement with subsequent visits, it is important for childhood obesity treatment centers, including our own, to determine ways our healthcare providers, administration, clinic procedures and policies and financial issues can be addressed to ensure the availability of consistent follow-up appointments.

### Strengths

Although there are several important limitations described above, our research does offer new insight into longitudinal changes in QOL and PHQ9A for obese and severely obese youth in treatment. First, we chose to track both physical and psychosocial outcomes over time. Many longitudinal efforts only measure weight-based variables (BMI, BMI z-score, or nutrition/physical activity behaviors), and neglect psychosocial variables. When researchers do include psychosocial variables, they often only use one variable, which is most commonly depression. Secondly, published longitudinal research has typically been conducted in structured large-scale settings which allow for the controlling of appointment scheduling at specific time intervals either monthly as indicated in the expert recommendations for Stages 3 and 4 treatment [4] or based on specific behavioral treatment protocols (i.e., 3 months, 6 months, 12 months, etc.). Our study could not control for these factors and instead represents a real-world rural outpatient clinical retention effort for overweight youth and their families. Even without being able to schedule appoints on a routine basis, we found that QOL, PHQ9A and BMI z-score were positively affected over time for youth in our sample. This brings up an important point about clinical versus statistical significance. Clinically, for youth who have been gaining weight over time, decreases in weight gain, leveling of current weight, and subsequent weight loss are all considered successes. The results of our study show that those participants who continued through a second and third visit had leveling of BMI z-score or slight declines, which is clinically significant, even though it only just had statistically significance.

### Implications

In order to address the different ways obesity affects both physical and psychosocial variables for youth and their family, these findings suggest benefit to using brief validated measures, such as the PedsQL to explore youth and caregiver perceptions of youth’s QOL and the PHQ9A to assess depression in teens and caregivers. In addition, there may be additional benefit to clinicians and researchers tracking youth longitudinally throughout treatment to investigate the relationship between youth’s QOL and those who level or decline in BMI verses those who increase or gain; specifically, to help determine if there is a certain QOL threshold that youth may reach before they begin to show signs of weight loss. Addressing both physical and psychosocial variables within medical treatment, as now recommended in care recommendations, is one way to incorporate these factors into the complex care of obese youth and their families and hopefully enhance overall success of treatment.

## Conclusions

Overall, across three visits our results indicated youth’s BMI z-score decreased slightly, their QOL significantly increased, and teen depression level improved. Likewise, caregivers’ perception of their youth’s QOL increased across three visits. Youth in our study, despite their BMI z-score, had significant improvements in QOL. Given that the majority of our sample was obese or severely obese, this is an important point that contests previous research which suggests that the most overweight youth have the most significantly impaired QOL. Additionally, after adjusting for time, improvement in teen depression was strongly related to QOL improvement which punctuates the value in assessing for depression simultaneously with a QOL inventory.

## Abbreviations

PHWRTC: Pediatric healthy weight research and treatment center; QOL: Quality of life; BMI: Body mass index.

## Competing interests

The authors have no competing interests or financial conflicts to disclose.

## Authors’ contributions

KP led the conception, design, data collection, writing of the manuscript, and participated in data analysis. SL, AL, and DC contributed to the conception, design, writing and revising the manuscript for submission. AI led the data analysis, and assisted with writing the results of the manuscript. All authors read and approved the final manuscript.
